# The Neural Bases of Drawing. A Meta-analysis and a Systematic Literature Review of Neurofunctional Studies in Healthy Individuals

**DOI:** 10.1007/s11065-021-09494-4

**Published:** 2021-03-16

**Authors:** Simona Raimo, Gabriella Santangelo, Luigi Trojano

**Affiliations:** Department of Psychology, University of Campania ‘Luigi Vanvitelli’, Caserta, Italy

**Keywords:** Meta-analysis, Constructional tasks, Drawing, Parietal lobes

## Abstract

Drawing is a multi-component process requiring a wide range of cognitive abilities. Several studies on patients with focal brain lesions and functional neuroimaging studies on healthy individuals demonstrated that drawing is associated with a wide brain network. However, the neural structures specifically related to drawing remain to be better comprehended. We conducted a systematic review complemented by a meta-analytic approach to identify the core neural underpinnings related to drawing in healthy individuals. In analysing the selected studies, we took into account the type of the control task employed (i.e. motor or non-motor) and the type of drawn stimulus (i.e. geometric, figurative, or nonsense). The results showed that a fronto-parietal network, particularly on the left side of the brain, was involved in drawing when compared with other motor activities. Drawing figurative images additionally activated the inferior frontal gyrus and the inferior temporal cortex, brain areas involved in selection of semantic features of objects and in visual semantic processing. Moreover, copying more than drawing from memory was associated with the activation of extrastriate cortex (BA 18, 19). The activation likelihood estimation coordinate-based meta-analysis revealed a core neural network specifically associated with drawing which included the premotor area (BA 6) and the inferior parietal lobe (BA 40) bilaterally, and the left precuneus (BA 7).

These results showed that a fronto-parietal network is specifically involved in drawing and suggested that a crucial role is played by the (left) inferior parietal lobe, consistent with classical literature on constructional apraxia.

## Introduction

Drawing is a unique human skill, and a sign of the evolution of the human brain to its sophisticated symbolic and communicative abilities (Cavanagh, 2005; Trojano et al., 2009). In contrast to simple motor tasks, drawing implies complex integration of a series of systems to transform a mental representation into a series of motor commands (Smith, 2009). Therefore, the study of drawing can provide deep insight for understanding the relationships between brain functioning and human information processing (Neistadt, 1993).

Because of its cognitive complexity, drawing plays an important role in clinical neuropsychology, as it is sensitive to several cognitive defects in patients with definite or suspected brain pathologies (see Gainotti & Trojano, 2018, for review). At the beginning of the twentieth century, Kleist (1934) and Strauss (1924) focused their attention on disorders of drawing, and suggested that a specific mental process was implied in putting together simple units so as to form complex figures or patterns, as in drawing and in building two- or three-dimensional structures, collectively considered as constructional tasks. On the basis of accurate observations on brain-lesioned patients, Kleist and Strauss proposed that this specific process would constitute a link between the visual-spatial functions and the kinesthetic engrams implied in manual activity, and would be localized in the left posterior parietal lobe, particularly in the left angular gyrus (for a historical review, Trojano, 2020).

After several theoretical and anatomo-clinical criticisms of the original proposal, for example, suggesting a predominant role of the right hemisphere in visuoconstructional tasks (Piercy et al., 1960), modern quantitative studies on focal brain-damaged patients demonstrated that many brain structures contribute to performance on drawing tasks. For instance, Chechlacz et al. (2014) showed that different lesions in the two hemispheres were significantly associated with selected facets of copying complex figures. In particular, overall accuracy correlated with lesions of the subcortical nuclei in the right hemisphere, whereas spatial errors correlated with a wide range of brain lesions, including the insula and inferior temporal gyri, in both the right and the left hemisphere, and the precuneus in the left hemisphere. These findings were clearly consistent with the idea that drawing depends on wide interconnected neural networks but suggested that these networks would not include the left inferior parietal region. This conclusion was confirmed by a re-analysis on a subset of the same sample (Chen et al., 2016). The multi-component nature of drawing tasks has been supported by a further quantitative study on focal brain-lesioned patients without clinically relevant right paresis or limb apraxia (Biesbroek et al., 2014). This study reported that poor performance in copying a complex figure was associated with lesions in the right superior parietal lobule, angular gyrus and middle occipital gyri, in the lack of a visuospatial perceptual impairment.

Therefore, the above studies did not provide consistent findings, and above all, did not support the original proposal of an important role for the left inferior parietal lobule in drawing tasks. However, in interpreting these findings it is important to take into account that many cognitive processes, including visual perception, visuospatial attention, high-level motor control, cognitive and sequence planning among others, contribute to performance in copying complex figures, and that most often brain-damaged patients are affected by cognitive impairments that hamper addressing the specific neural correlates of the drawing tasks. One strategy to overcome such difficulty is to adopt statistical procedures aimed at discounting the influence of non-specific cognitive impairments on drawing performance (e.g., Chen et al., 2016). Nonetheless, there is no guarantee that linear or non-linear algorithms could control for the effects of non-specific cognitive defects on drawing.

Another strategy to address the issue of the neural underpinnings of drawing and the possible role of the left inferior parietal lobe could be to investigate the neural correlates of drawing in healthy individuals by means of functional neuroimaging techniques. Indeed, in recent years several studies investigated drawing in healthy individuals by means of positron emission tomography (PET) or functional magnetic resonance imaging (fMRI). However, such studies had to tackle the relevant artefacts induced by hand and arm movements. For this reason, the majority of experimental paradigms included drawing-related tasks, rather than actual drawing, and only very recently fMRI-compatible graphic tablets allowed the analysis of real drawing. Another problem related to neurofunctional investigation of drawing is the choice of the control task(s) that could allow singling out the sensorimotor and cognitive components not specifically related to drawing. The difficulties inherent to the neurofunctional approach yielded a heterogeneous pattern of studies, employing different kinds of active condition, ranging from simulating drawing by means of finger movements in the air (Ino et al., 2003), to copying cartoons (Miall et al., 2009) and control tasks ranging from visual fixation (Simos et al., 2017) to trace the same lines to be copied in the active task (Ogawa & Inui, 2009).

Faced with this complex situation, a meta-analytic approach could identify the core neural structures related to drawing. Indeed, one recent meta-analysis (Yuan & Brown, 2015) on neurofunctional studies on drawing and handwriting reported i) a common activation in motor areas such as motor cortex, frontal eye field, supplementary motor area, cerebellum, putamen, ii) activation in posterior parietal cortex, involved in the visual guidance of hand movement and the formation of visual shapes, and iii) a specific activation of the ventral part of the left posterior parietal lobe, involved in the reproduction of pictures (drawing) but not of letters (writing). However, Yuan and Brown (2015) meta-analysis included a smaller number of studies compared to that recommended from the current guidelines proposed by Eickhoff et al. (2016) to perform an ALE meta-analysis, and some of the selected studies (Jueptner et al., 1996; Kawashima et al., 2000; Lerner et al., 2004; Ogawa et al., 2007; Seitz et al., 1997; Suchan et al., 2002) did not assess the neural correlates of constructional abilities, as they employed tasks such as drawing a single straight line, tracing curves, or connecting encircled numbers in ascending order.

On this basis, the present systematic review, complemented by a meta-analytic approach, aimed at identifying the specific neural bases of drawing, intended as the ability to producing images or figures composed of multiple parts in given spatial relationships with each other, by disentangling the brain regions involved in drawing from those activated by other fine motor activities (e.g. tapping, tracing lines, writing) and taking into account the type of drawn stimulus (figurative, geometric, or abstract).

## Materials and Methods

### Literature Search and Selection Criteria

**Fig. 1:**
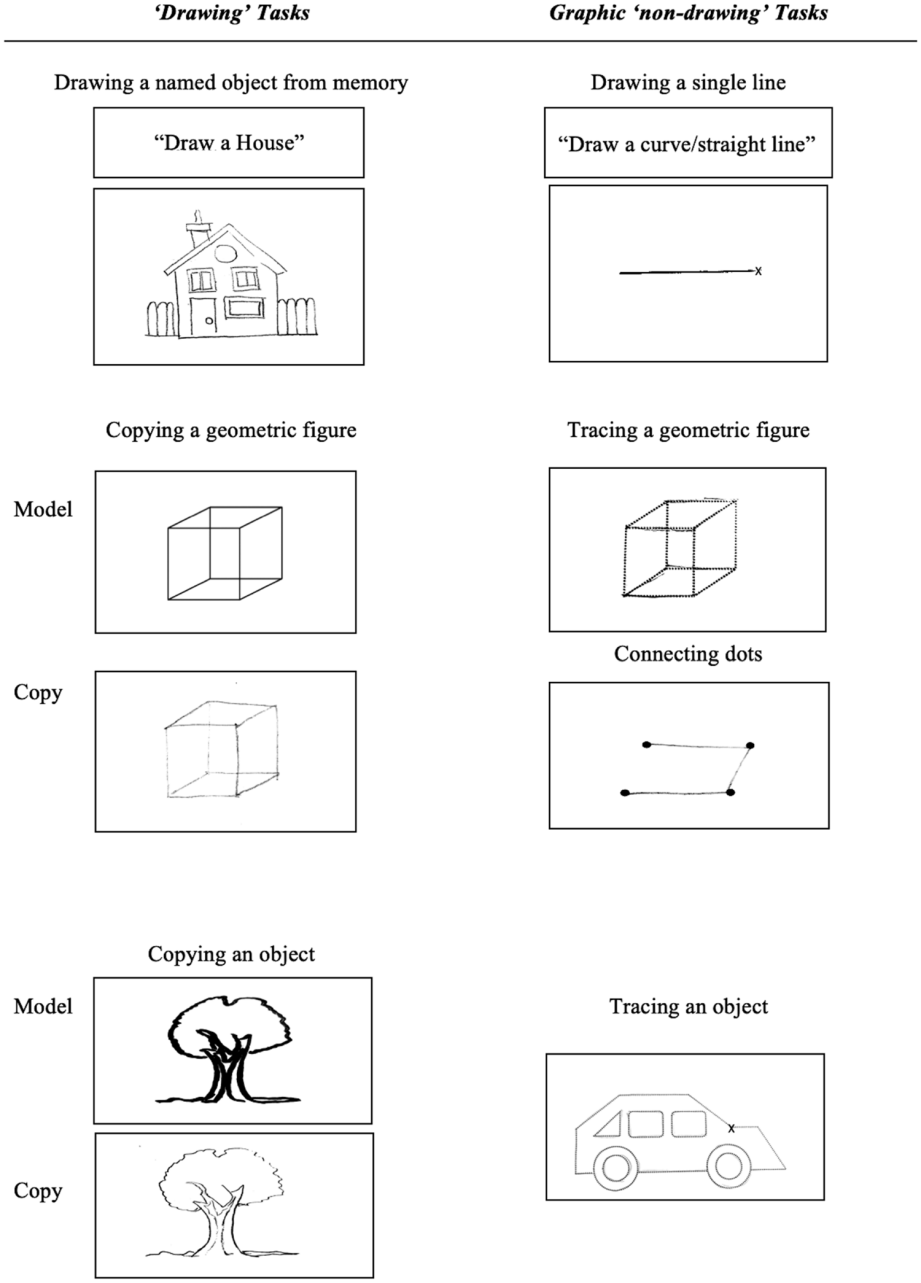
Instances of ‘drawing’ tasks, that were the focus of the study, and of graphic ‘non-drawing’ tasks that were only considered when compared to ‘drawing’ tasks proper; some studies investigated imagined rather than actual drawing to avoid artifacts related to hand and arm movements

A systematic approach combining different bibliographic medical databases (PubMed, Scopus, and Web of Science) together with a previous meta-analysis (Yuan & Brown, 2015) was adopted to obtain a comprehensive paper selection of the existing neuroimaging literature on drawing in healthy individuals. The following keywords in appropriate combinations were used to identify articles: (“drawing” OR “copying” OR “tracing” OR “constructional”) AND (“neuroimaging” OR “functional magnetic resonance” OR “fMRI” OR “positron emission tomography” OR “PET”). We intentionally used a larger number of keywords compared to the previous meta-analysis (Yuan & Brown, 2015) to increase the likelihood of identifying relevant articles. The process of selecting eligible articles was performed according to the Preferred Reporting Items for Systematic Review and Meta-Analysis (PRISMA) statement (Moher et al., 2009). Two authors (S.R. and L.T.) screened all titles and abstracts in electronic databases. Inclusion in the present meta-analysis required that articles i) had the full-text published in English peer-reviewed journal, ii) reported original data obtained from groups of healthy adults (not reviews or studies on patients with brain diseases or peripheral motor or sensory impairments), iii) reported results from brain imaging techniques (fMRI or PET), iv) performed whole-brain general linear model analyses since coordinate-based meta-analyses look for spatial convergence across experiments (thereby excluding studies reporting region-of-interest analyses, partial brain coverage, or small volume corrected results; Eickhoff et al., 2012; Müller et al., 2018), v) reported results in relation to a stereotactic coordinates system, and provided coordinates of activation foci either in the Montreal Neurological Institute and Hospital (MNI) or Talairach reference space, vi) focused on drawing tasks, intended as tasks where participants were required to produce a figure with its component parts in their correct spatial relationships in order to form a coherent and organized whole, following the classical definition of constructional abilities (Critchley, 1953; Kleist, 1934; Strauss, 1924). In relation to this last criterion, we included studies using (actual or imagined) ‘drawing’ tasks such as drawing a named object from memory (e.g. a watch, a face, a house, a clock, and so on), and copying simple or complex geometric figures (e.g. a triangle, a square, a cube, the Rey-Osterrieth figure) or objects (e.g. a face, a car, a book illustration, and so on). Studies that only employed tasks such as drawing single curve or straight lines, tracing figures (i.e. following the outline of a visual stimulus), or connecting dots were not included in the study, unless they contrasted any of these graphic ‘non-drawing’ tasks with ‘drawing’ tasks proper. Example of ‘drawing’ and of graphic ‘non-drawing’ tasks are provided in Fig. 1.

For each selected article we assigned a Quality Score according to a modified version of Downs and Black’s checklist for quality assessment (Downs & Black, 1998) as reported by Ayoub et al. (2018). The total scores range from 0 to 20, with higher scores meaning good external and internal validity. Two authors (S.R. and L.T.) evaluated each study independently, and disagreements were discussed and decided by consensus. Based on the recommended best practice guidelines for neuroimaging meta-analyses (Müller et al., 2018), we pre-registered the study on the PROSPERO platform (https://www.crd.york.ac.uk/PROSPERO/; registration number: CRD42020155472).

### Data Extraction

Data from each paper were classified according to the type of comparison investigated, namely, ‘drawing vs non-motor conditions’ (e.g. rest, fixation, or passive viewing) or ‘drawing vs other motor conditions’ (e.g. tapping, writing, or ‘non-drawing’ tasks) and the type of stimulus to be drawn, figurative, geometric, or abstract (non-sense). For each primary study the following relevant information was extracted i) the statistically significant brain coordinates (as determined by an α level of 0.05; two-tailed), classified according to the space in which they were reported (Talairach or MNI), ii) number of participants and their characteristics, including age, gender distribution, education, handedness, iii) type of comparison: 'drawing vs non-motor conditions', 'drawing vs other motor conditions', iv) type of figure that the participant had to draw, figuratives, geometrics, abstracts, and v) physical device used for drawing, pencil, mouse, tablet.

### Activation Likelihood Estimation Meta-analysis and Systematic Review

For a quantitative assessment of inter-study convergence we performed a coordinate-based meta-analysis (CBMA; Müller et al., 2018) using the Activation likelihood estimation (ALE) algorithm (Eickhoff et al., 2009; Turkeltaub et al., 2002) running under GingerALE software (http://brainmap.org/ale/) version 3.0.2. (Eickhoff et al., 2017). This algorithm, using input foci (e.g., brain coordinates) from multiple experiments, allows to identify significant convergence among reported coordinates in experiments that is higher than expected from a random distribution of foci (for further details on the ALE method please refer to the original publications of Eickhoff et al., 2012; Eickhoff et al., 2009; and Turkeltaub et al., 2012; Tahmasian et al., 2019). For each ALE calculation, significance was tested using 1000 permutations with a cluster forming threshold at voxel-level p < 0.001 (Eickhoff et al., 2016), and to provide an appropriate compromise between sensitivity and specificity, significance was corrected with a cluster-level family-wise error threshold of p < 0.05 (cFWE; Eickhoff et al., 2016), as in previous meta-analytic studies (Papitto et al., 2020; Teghil et al., 2019).

The ALE results, as significant clusters of convergence with clear anatomical properties (x, y, and z location in MNI), number of voxels, and p‐value, were automatically exported from GingerALE as NIfTI files (Belyk & Brown, 2014; Garrison et al., 2013; Zaccarella et al., 2017), overlaid onto a standard MNI template of MRIcroGL (http://www.mccauslandcenter.sc.edu/mricrogl/). To control for negative impact on the statistical validity of the meta-analysis, when two experiments were presented in the same paper (Turkeltaub et al., 2002) they were considered as one if the group of participants was the same, or different if the two groups of participants differed (Müller et al., 2018). Before performing CBMA, all coordinates extracted from each primary study and reported in Talairach space were transformed into MNI coordinates using the built-in icbm2tal function implemented in the GingerALE toolbox (Laird et al., 2005; Lancaster et al., 2007) and available at https://www.brainmap.org/ale.

Since the number of studies was not sufficient to achieve sufficient statistical power (i.e. a sample size of at least 17–20 experiments that should be used to guarantee the validity of ALE results; see Eickhoff et al., 2016; Müller et al., 2018), we conducted a systematic review on neuroimaging evidence focusing on the type of comparison investigated (e.g. ‘drawing vs non-motor conditions’ or ‘drawing vs other motor conditions’) and the type of stimulus (figurative, geometric, or abstract).

## Results

### Literature Search

**Fig. 2:**
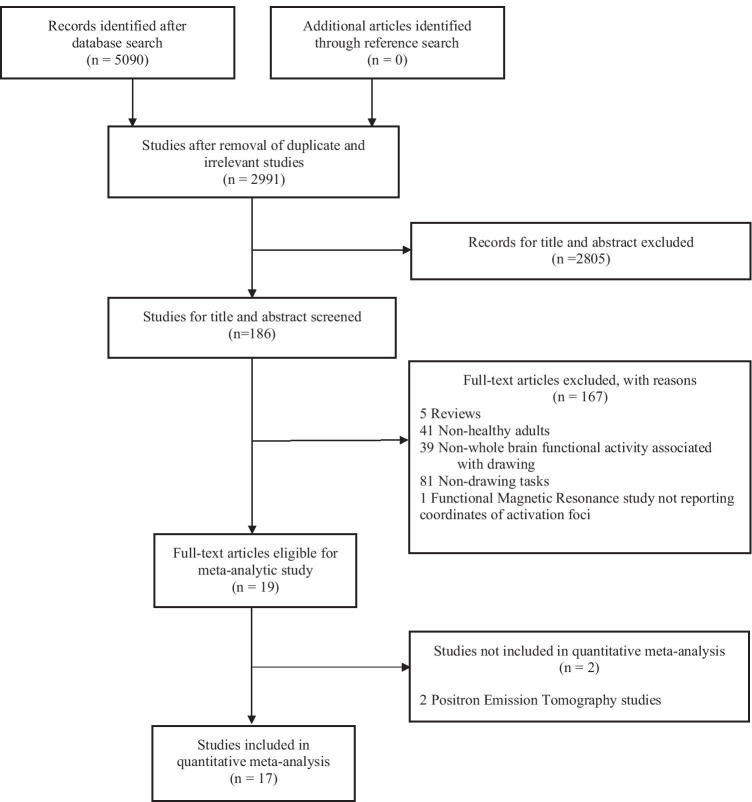
Flowchart of the selection process of primary studies

Figure 2 depicted the flow of the selection process based on PRISMA statement. The initial search identified 5090 articles. After removing duplicates, we obtained 2991 articles. Out of these, 2805 articles were excluded on the basis of title and abstract. After the full-text assessment, one study using functional magnetic resonance (fMRI) was excluded as it did not report the stereotaxic coordinates (Farias et al., 2006), whereas 19 articles met inclusion criteria and were considered eligible. As only 2 studies used Positron Emission of Tomography (PET; Seitz et al., 1997; Thut et al., 1997), we decided to perform a meta-analysis considering fMRI studies only. Detailed information concerning participant’s characteristics, experimental paradigm, and neuroimaging techniques of the 17 fMRI studies considered for the current quantitative meta-analysis are reported in Table 1. No eligible study compared healthy adults with brain-lesioned patients.

**Table 1:** Demographical, technical and methodological details of 17 studies included in the meta-analysis

Study	Number of subject	Age	Education	Handness	Process	Device	Content	Comparison analysis	Template	QS (0/20)
Ellamil et al., 2012	15 (6 M; 9F)	22.14 ± 2.25	NR	Right-handed	Book cover illustrations drawing	Pencil on table paper	Figuratives	Drawing > evaluation process	MNI	15
Ferber et al., 2007*	12 (4 M; 8F)	27.8 ± 3.9	NR	Right-handed	Objects drawing, copying and tracing	Pencil on tablet paper	Figuratives	Drawing and Copying > Tracing	Talairach	11
Gowen & Miall, 2007*	10 (5 M; 5F)	22.2	NR	Right-handed	Combinations of shapes drawing	Covert Production; Pencil on paper	Geometrics	Drawing > fixation Drawing > tracing	MNI	11
Habas & Cabanis, 2008	7	25	NR	Right-handed	Geometric figures drawing	Index finger in the air	Geometrics	Drawing > rest	MNI	10
Harrington et al., 2007*	11 (4 M; 6F)	37.1	NR	Right-handed	Objects drawing	Covert production	Figuratives	Drawing > rest Drawing > writing	Talairach	11
Harrington et al., 2009	8 (1 M; 7F)	32.8	NR	Right-handed	Objects and non-objects drawing	Covert production	Figuratives; Abstracts	Drawing > rest	Talairach	10
Ino et al., 2003*	18 (11 M; 7F)	32.3 ± 6.8	NR	Right-handed	Clock drawing	Index finger on plastic board	Figuratives	Clock drawing > line drawing	MNI	11
Makuuchi et al., 2003	17 (17 M)	23.7	NR	Right-handed	Objects copying	Index finger in the air	Figuratives	Copying and naming > naming	MNI	11
Miall et al., 2009	13 (8 M; 5F)	18–50	NR	Right-handed	Faces drawing and copying	Pencil pad	Figuratives	Drawing and copying > mental arithmetic condition	MNI	11
Miall et al., 2014	14 (6 M; 8F)	30	NR	Right-handed	Faces or abstract objects copying	Pencil on tablet paper	Figuratives; Abstracts	Copying faces > copying abstract objects	MNI	13
Ogawa & Inui, 2009*	28 (15 M; 13F)	25	NR	Right-handed	Curve patterns drawing and copying	Mouse pad	Geometrics	Copying > tracing	MNI	13
Planton et al., 2017	16 (8 M; 8F)	25.3 ± 6	NR	Right-handed	Object drawing	Pencil on tablet paper	Figuratives	Drawing > rest	MNI	13
Potgieser et al., 2015*	16 (7 M; 9F)	26.8 ± 9.8	NR	Right-handed	Geometric figures drawing	Pencil on paper	Geometrics	Drawing > tapping	MNI	13
Schaer et al., 2012*	20 (20 F)	22.45 ± 3.07	NR	Right-handed	Faces copying	Pencil on paper	Figuratives	Copying > passive viewing Copying > tracing	MNI	12
Simos et al., 2017	21 (6 M; 15F)	34.5 ± 7.4	NR	Right-handed	Triangle drawing	Index finger on board; Cover production	Geometrics	Drawing > fixation	MNI	11
Talwar et al., 2019	33 (14 M; 19F)	Median: 71	Median: 16	Right-handed	Clock drawing	Pencil on tablet paper	Figuratives	Drawing > fixation	MNI	13
Yuan and Brown., 2014	15 (6 M; 9F)	25	NR	Right-handed	Geometric figures drawing and copying	Mouse tablet	Geometrics	Drawing and copying > fixation	Talairach	12

^*^studies that compared ‘drawing vs non-motor action condition’, *MNI* montreal neurological institute and hospital, *NR* not reported

### General Meta-analysis

**Fig. 3:**
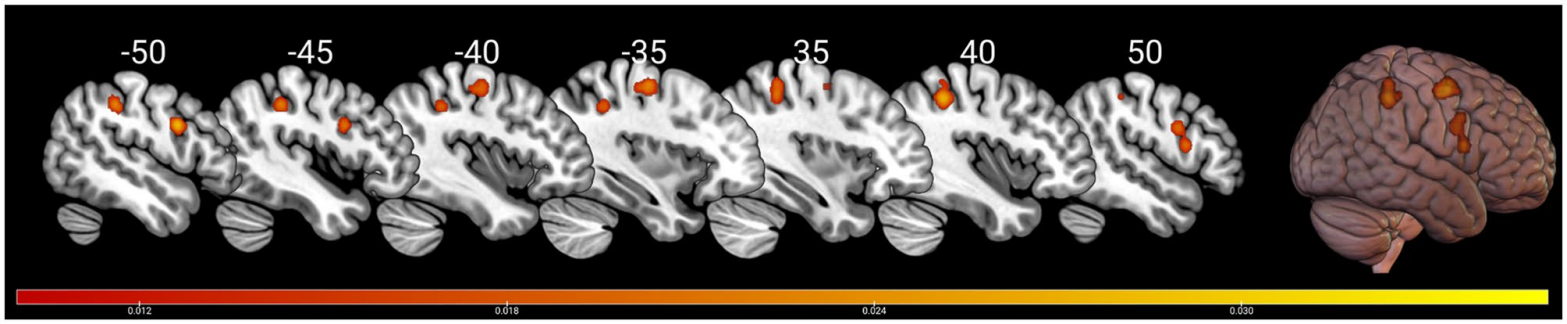
Results of the general ALE meta-analysis. Representative slices are displayed on 2D sagittal sections, with *x* MNI coordinate shown on the top of each, and on a 3D render. The color bar indicates activation likelihood estimation (ALE) values

The 17 studies totaled 274 healthy participants with a mean age of 27 years (range 18–85 years). All participants were right-handed, 97 participants (35.4%) were involved in drawing geometric figures; 140 participants (51%) were involved in drawing figurative images (such as a clock, faces, or common objects), and 37 participants (13.5%) were involved in drawing of abstract images. The general ALE analysis revealed clusters of activation in both hemispheres (Fig. 3 and Table 2). In particular, we found clusters of activation in the bilateral premotor area (BA 6) and inferior parietal lobe (BA 40), and in the left precuneus and superior parietal lobe (BA 7).

**Table 2:** Results of general ALE meta-analysis. For each cluster region label, hemisphere, cluster size (mm^3^), ALE value, p and z values, and MNI coordinates are provided

Cluster	Region	Hemisphere	Volume (mm^3^)	ALE value	*p*	Z	x	y	z
1	Premotor area/Supplementary motor area (BA 6)	LH	3712	0.024 0.024 0.020	< 0.001 < 0.001 < 0.001	4.794 4.721 4.208	-26 -38 -6	-6 -16 -10	54 52 60
2	Inferior Parietal Lobe (BA 40)	RH	1904	0.027 0.020 0.015	< 0.001 < 0.001 < 0.001	5.251 4.231 3.484	38 34 48	-40 -40 -32	48 56 48
3	Superior Parietal Lobe/Precuneus (BA 7)	LH	1776	0.024 0.024	< 0.001 < 0.001	4.733 4.726	-18 -22	-68 -64	54 56
4	Premotor area (BA 6)	RH	1696	0.024 0.021 0.020	< 0.001 < 0.001 < 0.001	4.751 4.367 4.205	52 52 56	12 6 8	14 26 32
5	Inferior Parietal Lobe (BA 40)	LH	1600	0.026 0.019 0.019	< 0.001 < 0.001 < 0.001	5.033 4.115 4.082	-48 -36 -40	-36 -44 -42	42 40 40

*LH* left hemisphere, *RH* right hemisphere

### Drawing Versus Non-motor Conditions

Twelve studies (Ellamil et al., 2012; Gowen & Miall, 2007; Habas & Cabanis, 2008; Harrington et al., 2007, 2009; Makuuchi et al., 2003; Miall et al., 2009; Planton et al., 2017; Schaer et al., 2012; Simos et al., 2017; Talwar et al., 2019; Yuan & Brown, 2014) investigated brain activation comparing drawing tasks with resting-state or non-motor conditions (e.g. mental arithmetics, fixation, passive viewing), and totaled 186 healthy participants, with 96 participants (51%) involved in drawing of figurative images (book cover illustrations, common objects, faces, and a clock). In particular, three of these studies (Makuuchi et al., 2003; Schaer et al., 2012; Yuan & Brown, 2014) investigated the neural bases of drawing by copying tasks, and other three studies (Harrington et al., 2007, 2009; Simos et al., 2017) employed imagined drawing. Since the number of studies was low (see Eickhoff et al., 2016), we did not perform an ALE meta-analysis but systematically reported the main data of the included studies (see Table 3).

**Table 3:** Overall results of the systematic review. Total number and percentage of studies reporting significant activation in each brain region

Region	Drawing versus non-motor conditions		Drawing versus other motor condition		Drawing figurative images		Drawing geometric images	
	Numbers of studies	Percent of studies	Numbers of studies	Percent of studies	Percent of studies	Percent of studies	Number of studies	Percent of studies
*Frontal Lobe*								
L Inferior frontal gyrus (BA 44)	5/12	41.6%	-	-	10/11	90.9%	1/6	16.6%
R Inferior frontal gyrus (BA 44)	4/12	33%	2/7	28.5%	10/11	90.9%	-	-
L Premotor/Supplementary motor area (BA 6)	11/12	91.6%	7/7	100%	11/11	100%	6/6	100%
R Premotor/Supplementary motor area (BA 6)	7/12	58.3%	5/7	71.4%	11/11	100%	3/6	50%
L Precentral gyrus (BA 4)	10/12	83%	2/7	28.5%	5/11	45.4%	3/6	50%
*Insula*								
L Insular cortex (BA 13)	6/12	50%	1/7	14.2%	3/11	27.2%	1/6	16.6%
R Insular cortex (BA 13)	3/12	25%	-	-	-	-	1/6	16.6%
*Parietal Lobe*								
L Superior parietal lobe (BA 7)	11/12	91.6%	7/7	100%	11/11	100%	4/6	66%
R Superior parietal lobe (BA 7)	12/12	100%	5/7	71.4%	11/11	100%	6/6	100%
L Inferior parietal lobe (BA 40)	9/12	75%	6/7	85.7%	11/11	100%	5/6	83.3%
R Inferior parietal lobe (BA 40)	6/12	50%	3/7	42.8%	10/11	90.9%	6/6	100%
*Temporal Lobe*								
L Inferior temporal lobe (BA37)	5/12	41.6%	3/7	42.8%	10/11	90.9%	1/6	16.6%
R Inferior temporal lobe (BA37)	5/12	41.6%	3/7	42.8%	11/11	100%	1/6	16.6%
L Middle temporal gyrus	3/12	25%	2/7	28.5%	5/11	45.4%	-	-
R Middle temporal gyrus	3/12	25%	1/7	14.2%	4/11	36.3%	-	-
L Fusiform gyrus	3/12	25%	-	-	3/11	27.2%	-	-
R Fusiform gyrus	1/12	8.3%	1/7	14.2%	2/11	18.1%	-	-
*Occipital*								
L Inferior occipital gyrus (BA 19)	3/12	25%	2/7	28.5%	3/11	27.2%	-	-
R inferior occipital gyrus (BA 19)	1/12	8.3%	3/7	42.8%	4/11	36.3%	1/6	16.6%
*Cerebellum*								
L Lobuli IV-VI	5/12	41.6%	2/7	28.5%	4/11	36.3%	2/6	33.3%
R Lobuli IV-VI	10/12	83%	3/7	42.8%	5/11	45.4%	3/6	50%

*L* left, *R* right, *BA* broadmann area, - no study reported a significant activation

All twelve studies reported a significant activation in the superior parietal lobe (BA 7) bilaterally. Almost all of them reported a significant bilateral activation during drawing tasks in the premotor and supplementary motor area (BA 6; n = 11: Ellamil et al., 2012; Gowen & Miall, 2007; Habas & Cabanis, 2008; Harrington et al., 2007, 2009; Makuuchi et al., 2003; Miall et al., 2009; Planton et al., 2017; Schaer et al., 2012; Talwar et al., 2019; Yuan & Brown, 2014), the inferior parietal lobe (BA 40; n = 9: Ellamil et al., 2012; Habas & Cabanis, 2008; Harrington et al., 2007, 2009; Makuuchi et al., 2003; Miall et al., 2009; Planton et al., 2017; Talwar et al., 2019; Yuan & Brown, 2014), and the cerebellum (including the lobuli IV-VI; n = 10: Ellamil et al., 2012; Gowen & Miall, 2007; Habas & Cabanis, 2008; Harrington et al., 2009; Makuuchi et al., 2003; Miall et al., 2009; Schaer et al., 2012; Simos et al., 2017; Talwar et al., 2019; Yuan & Brown, 2014). Moreover, 10 studies found a significant activation in the left precentral gyrus (BA 4; Gowen & Miall, 2007; Habas & Cabanis, 2008; Harrington et al., 2007, 2009; Makuuchi et al., 2003; Miall et al., 2009; Planton et al., 2017; Schaer et al., 2012; Talwar et al., 2019; Yuan & Brown, 2014). The same brain pattern of activation was found in the three studies (Harrington et al., 2007, 2009; Simos et al., 2017) using imagined drawing, in addition to a common activation in the bilateral inferior frontal gyrus (BA 44) and insular cortex (BA13).

### Drawing Versus Other Motor Conditions

Seven studies (Ferber et al., 2007; Gowen & Miall, 2007; Harrington et al., 2007; Ino et al., 2003; Ogawa & Inui, 2009; Potgieser et al., 2015; Schaer et al., 2012) compared drawing tasks with other fine motor conditions (e.g. tracing, writing, line drawing), and totaled 115 healthy participants, 61 participants (53%) involved in drawing of figurative images (common objects, faces, and a clock), and 54 participants (46.9%) involved in drawing of geometrics figure. In particular, three of the studies (Ferber et al., 2007; Ogawa & Inui, 2009; Schaer et al., 2012) contrasted copying versus tracing and other two studies (Ferber et al., 2007; Miall et al., 2009) contrasted drawing from memory versus copying. Since the number of studies was low (see Eickhoff et al., 2016), we did not perform an ALE meta-analysis but systematically reported main data of the selected studies (see Table 3).

All studies found a significant activation in the left premotor and the supplementary motor area (BA 6) and the superior parietal lobe (BA 7). Almost all of these studies, but one (Schaer et al., 2012) found an activation of the left inferior parietal lobe (BA 40).

The three studies (Ferber et al., 2007; Ogawa & Inui, 2009; Schaer et al., 2012) in which copying was contrasted with tracing contours reported activation in the cuneus and the lingual gyrus (BA 18, 19) without clear hemisphere lateralization; the same pattern was reported in the three studies comparing copying with drawing from memory (Ferber et al., 2007; Miall et al., 2009; Yuan & Brown, 2014).

### Drawing of Figurative, Geometric or Abstract Images

Eleven studies investigated drawing figurative images (Ellamil et al., 2012; Ferber et al., 2007; Harrington et al., 2007, 2009; Ino et al., 2003; Makuuchi et al., 2003; Miall et al., 2009; Miall et al., 2014; Planton et al., 2017; Schaer et al., 2012; Talwar et al., 2019) in a total sample of 177 healthy participants. Among these, 5 studies (Makuuchi et al., 2003; Harrington et al., 2007, 2009; Ferber et al., 2007; Planton et al., 2017) required participants to draw common objects, 3 (Miall et al., 2009, 2014; Schaer et al., 2012) required participants to draw faces, and 2 (Ino et al., 2003; Talwar et al., 2019) required participants to draw a clock; only one study (Ellamil et al., 2012) required to draw book cover illustrations according to book descriptions. Since the number of studies was low (see Eickhoff et al., 2016), we did not perform an ALE meta-analysis but systematically reported main data of the included studies (see Table 3).

All studies reported a significant activation in the bilateral premotor cortex (BA 6), the inferior (BA 40) and superior (BA 7) parietal lobe, and the inferior temporal lobe (BA 37). Almost all studies, but one (Makuuchi et al., 2003) reported a significant activation of the bilateral inferior frontal gyrus (BA 44, 46). Moreover, when participants were required to draw a face, an activation of the bilateral fusiform gyrus was found.

Six studies investigated drawing geometric figures (Gowen & Miall, 2007; Habas & Cabanis, 2008; Ogawa & Inui, 2009; Potgieser et al., 2015; Simos et al., 2017; Yuan & Brown, 2014) in a total sample of 82 healthy participants. Almost all of these required participants to draw single geometric shapes, such as square or triangle or circle, whereas two studies (Gowen & Miall, 2007; Yuan & Brown, 2014) required to draw a pattern of geometric shapes in a specific order. Since the number of studies was low (see Eickhoff et al., 2016), we did not perform an ALE meta-analysis but systematically reported main data of the selected studies. All studies reported a significant activation during drawing geometric figures in the premotor and supplementary motor areas (BA 6), and in the inferior (BA 40) and superior parietal lobe (BA 7), without clear hemisphere lateralization. Almost all these studies, but one (Ogawa & Inui, 2009), showed a significant activation of the right cerebellum (including crus IV-VII).

Two studies (Harrington et al., 2009; Miall et al., 2014) investigated drawing of abstract images in 21 healthy participants and reported that drawing familiar objects compared to drawing non-objects was significantly associated with the activation of the inferior temporal and fusiform gyri as well as the inferior frontal regions (pars opercularis and pars triangularis).

## Discussion

In the present study we addressed the specific neural bases of drawing. A previous meta-analysis on drawing and handwriting (Yuan & Brown, 2015) included six tracing and line drawing studies (Jueptner et al., 1996; Kawashima et al., 2000; Lerner et al., 2004; Ogawa et al., 2007; Seits et al., 1997; Suchan et al., 2002) and studies using a voxel-wise false discovery rate control method, although this last procedure has low sensitivity and an increased risk of finding spurious clusters (Eickhoff et al., 2016). On this basis, we decided to conduct a systematic review complemented by a meta-analytic approach in which we: i) included more recent studies (Ellamil et al., 2012; Habas & Cabanis, 2008; Miall et al., 2014; Planton et al., 2017; Potgieser et al., 2015; Simos et al., 2017; Talwar et al., 2019; Yuan & Brown, 2014), ii) considered only studies in which the main task required participants to produce figures composed of multiple parts in given spatial relationships, following the classical perspective on constructional abilities (Critchley, 1953; Kleist, 1934; Strauss, 1924), whereas studies requiring tracing or drawing single lines were included only when these graphic motor tasks were contrasted with drawing proper, and iii) adopted a solid meta-analytic approach, the voxel-level cluster forming threshold of p < 0.001 and a cluster-level threshold of p < 0.05 (Eickhoff et al., 2016; Müller et al., 2018). Our meta-analytic results showed a core neural network, consisting of the supplementary motor area (BA 6) and the inferior parietal lobe (BA 40) bilaterally, and of the left precuneus (BA 7), specifically associated with drawing. These results were partly in line with the meta-analytic results of Yuan and Brown (2015), but only the brain regions playing an important role in the activity of combining simple elements for the purpose of building spatially determined shapes have been identified here, thus singling them out from the wide fronto-parietal network involved in other graphic activities, such as writing (Yuan & Brown, 2015).

In the search for the specific neural substrates of drawing, we split the papers selected for the present meta-analysis into two groups according to the type of the control task employed and examined the overall convergence between results in each group of studies. A substantial body of evidence demonstrated that ‘*drawing versus non-motor conditions*’ was mainly associated with the activation of the left primary motor cortex (BA 4) and the bilateral cerebellum, involved in motor control and execution of drawing tasks (He et al., 1995), beyond the activation of the ‘core’ fronto-parietal network including inferior parietal lobe, BA 40 and premotor areas BA 6. The analysis of studies addressing ‘*drawing versus other motor conditions*’ revealed that, by distinguishing the brain regions involved in drawing from those activated by other fine motor activities, the fronto-parietal network was generally activated on the left side of the brain. Moreover, all studies investigating copying versus tracing contours or drawing from memory reported activation in the cuneus and the lingual gyrus (BA 18, 19) without clear hemisphere lateralization.

These findings would confirm in healthy individuals what has been argued on the basis of clinical observations by early neurologists investigating constructional disabilities in brain-lesioned patients (Kleist, 1934; Strauss, 1924). Indeed, those authors proposed that the left inferior parietal lobe could be the brain region responsible for constructional abilities, that is, the brain region specifically implied in combining single elements in an integrated whole. The present evidence would thus suggest that the above-cited modern studies addressing anatomo-clinical correlates of constructional abilities (Biesbroek et al., 2014; Chechlacz et al., 2014; Chen et al., 2016) could not highlight the role of the left parietal lobe in drawing, as other brain lesions could determine cognitive defects contributing to, but not specifically responsible for, drawing impairments.

The finding that the inferior parietal cortex, particularly on the left side, is activated in drawing tasks is consistent with the plurality of processing streams linking it to the occipital and frontal lobes (Gainotti & Trojano, 2018). Within the well-known two-pathway hypothesis of visual processing (Milner & Goodale, 1993), the parietal lobe is crucial for encoding spatial relations to guide hand actions. Recent reappraisals of the hypothesis would suggest a division of the labor between the two pathways depending on contexts and tasks (Freud et al., 2016; Vaziri-Pashkam & Xu, 2017), but the basic notion remains that the inferior parietal lobe would contribute to constructing objects’ spatial representations, and the precuneus would be involved in maintaining the correct spatial relations of the object (spatial working memory) and planning visually guided hand movements (Bledowski et al., 2009).

As highlighted in a thorough review of anatomical and functional properties of the parietal lobes in monkeys and humans (Caminiti et al., 2015), the inferior parietal lobe would specifically carry out visual control of hand-object interaction for different kinds of hand actions within the ventral parieto-premotor stream, that was confirmed to be involved in drawing by the present findings. The activation in the lingual gyrus and cuneus during copying task can be ascribed to the involvement of these regions in visual processing and visual attention (Macaluso et al., 2000), cognitive processes required for comparing one’s own copy with the model, in agreement with single case studies (for instance, James et al., 2003) on patients with inferior occipital lesion who were unable to copy figures but could draw them from memory.

Finally, by dividing the selected studies as a function of the type of to-be-drawn stimulus, we found convergent evidence for the role of the bilateral inferior temporal lobe (BA 37) and inferior frontal gyrus (BA 44, 46) in drawing figurative images. According to the literature (Gainotti et al., 1983; Moore & Price, 1999; Thompson-Schill et al., 1997; Badre et al., 2005; Badre & Wagner, 2007) the inferior temporal cortex would be involved in visual semantic processing, whereas the anterior inferior frontal activation would be related to selection of specific semantic features of objects; this would explain their recruitment in drawing familiar objects.

In conclusion, the present meta-analysis and systematic review highlighted a specific involvement of a fronto-parietal network in drawing, including the inferior and superior parietal lobe, and the premotor cortex, coherently with literature on constructional apraxia (Gainotti & Trojano, 2018; Kaplan, 1988; Tranel et al., 2008). However, this study is not without limitations. Based on the inclusion criteria, seed‐based functional connectivity analysis in resting state fMRI, diffusion‐tensor imaging and spectroscopy studies were excluded from CBMA, thus reporting multiple spatially isolated clusters of coordinates. For this reason, the interpretation and conclusions of our study did not allow identification of networks of significant clusters coactivation. Moreover, the relatively limited pool of original research studies did not allow us to perform other quantitative investigations of the brain regions involved in drawing as a function of the type of the control task employed (‘*drawing versus non-motor conditions*’, or ‘*drawing versus non-motor conditions*’), the type of stimuli (figurative, geometrics, or abstracts), and of the type of process used (actual, imagined drawing, or copying). Nevertheless, these findings corroborate the hypothesis that the parietal lobe, and in particular its inferior part in the left hemisphere, plays a crucial role in the cognitive processes related to constructional activities. Further neuroimaging and experimental investigations are warranted to clarify the functions and cognitive processing streams within the parietal lobe devoted to process specific kinds of stimuli in the different constructional tasks.
